# Magnetic Source Imaging and Infant MEG: Current Trends and Technical Advances

**DOI:** 10.3390/brainsci9080181

**Published:** 2019-07-27

**Authors:** Chieh Kao, Yang Zhang

**Affiliations:** 1 Department of Speech-Language-Hearing Sciences, University of Minnesota, Minneapolis, MN 55455, USA; 2 Center for Neurobehavioral Development, University of Minnesota, Minneapolis, MN 55455, USA

**Keywords:** Magnetoencephalography (MEG), infant, cognitive development, source localization, equivalent current dipole (ECD), minimum norm estimation (MNE)

## Abstract

Magnetoencephalography (MEG) is known for its temporal precision and good spatial resolution in cognitive brain research. Nonetheless, it is still rarely used in developmental research, and its role in developmental cognitive neuroscience is not adequately addressed. The current review focuses on the source analysis of MEG measurement and its potential to answer critical questions on neural activation origins and patterns underlying infants’ early cognitive experience. The advantages of MEG source localization are discussed in comparison with functional magnetic resonance imaging (fMRI) and functional near-infrared spectroscopy (fNIRS), two leading imaging tools for studying cognition across age. Challenges of the current MEG experimental protocols are highlighted, including measurement and data processing, which could potentially be resolved by developing and improving both software and hardware. A selection of infant MEG research in auditory, speech, vision, motor, sleep, cross-modality, and clinical application is then summarized and discussed with a focus on the source localization analyses. Based on the literature review and the advancements of the infant MEG systems and source analysis software, typical practices of infant MEG data collection and analysis are summarized as the basis for future developmental cognitive research.

## 1. Introduction

**Figure 1:**
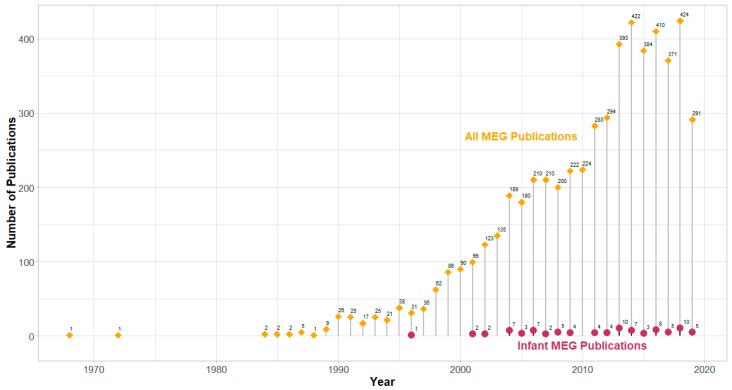
Number of magnetoencephalography (MEG) publications in the period of 1968–2019. Data were exported and replotted from the online PUBMED database (pubmed.gov; as of 21 July 2019) with search of target word “magnetoencephalography” in the title or abstract for “All MEG Publications”; and target words “magnetoencephalography” and “infant* or neonate* or newborn*” in the title or abstract for “Infant MEG Publications”.

Magnetoencephalography (MEG) is an important and completely non-invasive imaging technique for mapping functional brain activities in normal as well as pathological populations for basic research and clinical purposes. It uses special sensors called superconducting quantum interference devices (SQUIDs) to measure and visualize the exquisite online magnetic field changes from post-synaptic neuronal currents on the millisecond or sub-millisecond scale depending on the sampling rate of signal recording. Historically, these neuronal activities at the system level have been recorded noninvasively and studied with electroencephalography (EEG), which is much less expensive for instrumentation and measurement. EEG generally provides less precise localization of the cortical/subcortical sources compared with MEG in studies on early brain development [1] (but also see References [2,3] for successful EEG source localization examples). Unlike EEG, which is subject to signal smearing due to conductivity issues in the skull and scalp, the MEG signal is less susceptible to the heterogeneous anatomical structures and tissues between the sensors and the neuronal generators. When integrated with a three-dimensional head model built from an individual’s magnetic resonance imaging (MRI) scan, MEG becomes functional magnetic source imaging (MSI), which allows mapping the online millisecond-by-millisecond dynamics of mental activities with a spatial resolution on the order of millimeters [4]. In the first twenty years or so since Dr. Cohen’s seminal work in 1968 [5], MEG research and clinical applications were rather limited due to its availability, technicality required of both hardware and software, and cost. The last three decades have witnessed a steady surge of MEG publications with a plateau of approximately 400 per year in the last six years and a slow increase of infant MEG publications from 1996 (Figure 1). The increase of the MEG publications reflects fast growing interests and funding in the field of cognitive science and brain imaging propelled by technical advances in the digital computing world for real-time high-capacity processing and complex scientific visualization. With the increasing popularity of MEG, there have been a series of comprehensive scientific review papers [6,7,8,9,10,11] and textbooks/edited volumes [12,13,14,15,16,17,18] to introduce MEG to the scientific community, the medical field, and the wider audience in general. There have also been summary reports of MEG studies on special populations with various clinical conditions such as autism [19,20,21], epilepsy [22,23], schizophrenia [24,25,26], language impairment [27], dementia [28], dystonia [29,30], major depression disorder [31], obsessive-compulsive disorder [32], fibromyalgia syndrome [33], and other neurological and psychiatric disorders [8,34]. A guideline by Schwartz et al. [35] on pediatric MEG studies provides a detailed overview and some successful examples, reassuring the feasibility of using MEG to explore cognitive development in both typical and clinical populations.

Unlike fMRI, PET (positron emission tomography) and SPECT (single-photon emission computed tomography), which are secondary measures of metabolism over much longer time scales, MEG directly measures neuronal activities associated with various brain functions of interest. While MEG does not have capabilities to directly identify the biochemical, molecular, and genetic mechanisms for explaining brain structure and functions underlying mental and neurological disorders, it can be combined with various research tools to help determine the relationships between system-level and lower-level brain mechanisms and is well placed for multi-modal imaging. MEG and EEG can be simultaneously recorded with each providing complementary information about neuronal currents. Currently, the main clinical applications of MEG are for localizing epilepsy and pre-surgical mapping with improved surgical outcomes, and recent years have seen a steady increase of research interests and efforts in using MEG to characterize a broad range of neurological and psychiatric conditions for potential diagnostic purposes. For instance, by taking advantage of its hi-fidelity in magnetic source imaging with unprecedented accuracy on the individual subject basis, neuroscientists can systematically examine brain network interruptions that may lead to many clinical disorders. It has been shown that disruptions in brain connectivity are associated with Alzheimer’s [36] and autism [37]. 

One striking feature of the MEG literature is that infant (below 2-year-old) research represents less than 2% of the published studies. In comparison with EEG studies in infant research, the number of infant MEG publications appears to be very small, with a current ratio of 1:51 based on the PUBMED search results. Considering that the largest MEG manufacturer alone (Elekta) has sold over 100 MEG systems worldwide, it is surprising to see that the number of infant MEG studies is still far from reaching 100. Another noticeable feature is that despite the widely claimed advantages of magnetic source imaging over EEG, many infant MEG studies did not attempt or report source localization analysis to improve our understanding of the source loci of brain activations, hemispheric laterality, cortical distribution and connectivity patterns that illustrate the neuroanatomical basis of computational processes involved in the chosen experimental protocol. Therefore, at least some opportunities may have been missed to investigate neural processes with more details on audition, vision, motor control, memory, attention, emotion, language, and social cognition that could be disturbed in one way or another in mental disorders such as autism that have its origin early in life. The present review serves to illustrate the challenges, constraints, and potentials of MSI in the field of infant MEG research and suggest typical practices for future studies, which supplements the existing review summaries on infant MEG research [38,39,40]. For instance, a very recent review by Chen et al. [38] has covered the findings of most of the empirical infant MEG studies. In this summary report, we will focus more on the advancement of MEG source localization analysis and the technical challenges in infant MEG data acquisition and analysis. We also present the research questions and advances in source localization analysis, and some limitations and future directions of infant cognitive research using MEG. Additionally, we summarized the typical practices for infant MEG data acquisition and analysis based on the literature review.

### 1.1. Advantages of MEG Compared with fMRI and fNIRS in Developmental Studies

Localizing brain activities has long been associated with functional magnetic resonance imaging (fMRI) or functional near-infrared spectroscopy (fNIRS) as the two mainstream options for their high spatial resolution. For infant cognitive research, MEG measurement with advanced source localization analysis provides an alternative that can overcome some shortcomings of fMRI and fNIRS recordings. The MRI scanning for infant participants is generally undertaken while they are asleep. The noise and the constrained scanning environment limit the use of fMRI for investigating infants’ cognitive processing while they are awake. Furthermore, high-quality MRI data requires participants to stay still during the scanning sessions in order to get an accurate location of the changing hemodynamic response associated with the target cognitive task. Considering these factors, fMRI would not be an optimal tool to investigate neural mechanisms of cognitive processing in alert infants. 

On the other hand, fNIRS is comparatively more infant-friendly because it is silent and with slightly more motion-tolerant recording requirements; however, it has lower spatial resolution compared to fMRI [41]. Nonetheless, the near-infrared (NIR) lights can only reach the surface of the cortex, not the deeper brain structures. The low signal-to-noise ratio due to the NIR lights traveling through multiple layers before reaching the cortex also confines the studies to mostly use block designs to overcome this challenge. The vascular system on the cortical surface may also reflect the changes in heart rate or respiratory rate during the fNIRS recording, therefore contaminating the hemodynamic responses that are hypothesized to be contingent to the cognitive processes [42]. Another practical drawback is that the implementation of whole-head fNIRS would put all the weights of the optodes on infants’ heads. Furthermore, the depth that the NIR lights can detect would be limited due to the shorter distance between the light source and the detector, which constrains the exploratory whole-head measurement for infants.

The MEG measurement provides a more direct assessment of neuronal electric current changes in milliseconds rather than the indirect hemodynamic changes over seconds in fMRI and fNIRS. With zero noise, fast setup, and no applied magnetic field, radiation or injections, MEG is more comfortable and tolerable for the research subject with little safety risks. These features make MEG a more feasible tool for measuring awake infants’ neural registries of cognitive processing. The advanced source localization analysis using improved forward solutions and head models increases the spatial resolution of MEG measurement, making it a preferable tool for infant cognitive research. 

### 1.2. Current Technical Challenges in Developmental Cognitive Sciences with MEG

Despite the advantages over fMRI and fNIRS, infant MEG studies are not without technical barriers. The physical structures of an infant’s brain and head make it challenging to create proper head models for source localization analysis. First, neonate or older infant’s much smaller head size lengthens the distance between their brains and the MEG sensors in adult MEG helmets. The longer distance leads to weaker magnetic signals being measured, and the spatial resolution can be severely compromised due to the lower signal-to-noise ratios [43]. Moreover, the location of the infant’s head in the adult MEG helmet also affects the strength of the magnetic signals being recorded. For example, some studies placed infant’s right or left temporal side at the occipital position in the adult MEG helmet while they were sleeping [44,45,46]; whereas other studies let infant participants sit up like adult participants if the tasks required them to be awake [47,48]. The signal recorded at the side closest to the MEG helmet would be the strongest, and the weaker signals would be measured at the opposite side further away from the helmet [49]. Therefore, mapping out the uneven signals over the scalp and compensating for the inhomogeneity will need to be overcome for precise source localization. Another factor is that the yet to be closed fontanels and sutures in infants’ scalps lead to electrical leakage, and they further hinder accurate forward modeling. Even though the open fontanel and suture affect the MEG recordings less than electroencephalography (EEG) recordings and the inverse solutions are only weakly affected [1], the high variability in the location or size of the gaps across infants introduces inconsistency especially when their functional magnetic signals are mapped to a single-infant MRI template. Such problems can be mainly compensated by first acquiring individual structural MRI scans during infants’ sleep and then carrying out the functional MEG measurement. Another way is to use more realistic head models, which will be further discussed in the later sections.

One direct challenge for recording high-quality infant MEG signals comes from the movement-related artifacts. For example, experiments using adult MEG helmet to measure awake babies usually observe excessive head movements, which cause problems for averaging MEG responses across trials and further bias source localization prominently. Moreover, infants’ general head movement patterns are even significantly different from older children and adults [50]. Sometimes, excessive head and body movements are inevitable. For instance, pediatric epileptic patients cannot stay still for long in a MEG recording session. Despite the challenges, more and more pediatric epileptic studies adopted MEG as part of the pre-surgery evaluations in determining the focal sites of the epileptic activations due to the advancement of the head movement correction algorithms [51]. While head movements can be tracked and compensated if the dynamic changes of the head position are also recorded online, this is still a potential source of inaccurate estimation of the origins of the infant’s cognitive processing. Another potential artifact may stem from smaller body size, which results in stronger cardiac artifacts in infant MEG recordings than those from adults [52,53]. 

The current challenges in source determination of infant MEG measurement lie in the accuracy of the forward modeling and inverse solutions. The main goal of these models is to approximate infant brains and head structures and reconstruct the magnetic signals back to their neural generators. In order to have more accurate models, the anatomical structures of infants’ heads at different ages should be taken into account if templates of head models are to be used in MSI. Some earlier forward models adopted the parameters of adults’ anatomical brain and scalp layers, which were later proved to be detrimental to the precision of the forward modeling for infants [2]. Many of the recent studies aimed to advance the current forward modeling and inverse solutions for infant populations. For example, the use of realistic head model (Baby Connectome Project, [54]) with proper biophysical constraints (conductivity geometry) defined by individual anatomical or functional MRI images could help determine the contributions of different current sources to each MEG sensor measurement more accurately [1]. The rigid boundary element method (BEM) based on average infants’ scalp structures could also lead to a more precise estimation of how potential sources project the neurophysiological response to the sensors. Furthermore, MEG sensors are only sensitive to tangential sources, making source modeling more straightforward [55]. Even though there are still challenges for quality recording and room for improvement for the source localization analysis in infant MEG studies, being able to estimate the neural generators supporting cognitive tasks move this type of physiological measurements forward and beyond merely providing timing and strength of the neural activity at the sensor level.

Aside from the technical issues, the infant MEG setup and the experimental design also require special attention. The very first question is whether the infant participants need to be awake during the task. Measuring infants during sleep is easier than awake ones. However, some research questions can only be answered by measuring the evoked brain response from infants being actively engaged in the tasks. In such cases, making the magnetically shielded room infant-friendly and letting one of the parents stay close to the infant during the recording are necessary. Given the available experimental paradigms, visual studies are more challenging than auditory studies since most of the auditory studies used a passive listening paradigm. If the infant visual studies require infants’ eye fixation on the screen, then it is essential to develop carefully designed and attractive stimuli or properly insert some attention-getters in the visual presentation.

## 2. Topics of Infant Research Applying MEG Source Localization Analysis

**Figure 2:**
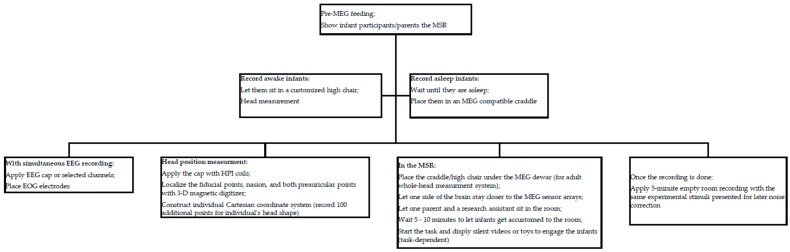
Summary of typical practices for infant MEG data acquisition. (MEG = magnetoencephalography; MSR = magnetically shielded room; EEG = electroencephalography; EOG = Electro-oculogram; HPI = head position indicators).

**Figure 3:**
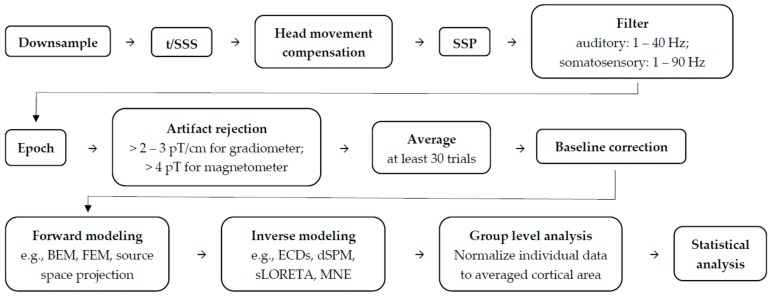
Summary of typical practices for infant MEG data preprocessing and analysis. The order of the MEG signals preprocessing varies from research topics. In order to organize the steps, we summarized the preprocessing procedures that include head movement compensation, which is an essential part for further source analysis in infant MEG studies. (also see [92,93]).

In order to discuss the progression of infant MEG source localization analysis, various related research topics are first presented and compared (see Table 1 for an overview). The age groups of the participants in the following studies mainly include newborns (or neonates, from birth to three months old) and infants (from three months old to 24 months old). Some longitudinal studies with fetal, child (from two years old to five years old), and adult (above 20 years old) participants are included in Table 1 but not further discussed in the main text. The adopted forward modeling and inverse calculation vary across research groups and research topics. Some widely used forward modeling includes a spherical head model or age-matched MRI template. The most frequently used inverse modeling is the equivalent current dipoles (ECDs); and the minimum norm estimate (MNE), standardized low-resolution brain electromagnetic tomography (sLORETA), exact low-resolution brain electromagnetic tomography (eLORETA), and dynamic statistical parametric mapping (dSPM) were also implemented in some studies. The current review will compare the source localization analysis in each infant cognitive research topic. Some other analysis approaches in infant MEG research not incorporating source analysis are also briefly summarized in this section. Typical practices of infant MEG data collection and analysis are then provided based on the summary report (see Figure 2 and Figure 3).

### 2.1. Auditory Processing

Over half of the infant MEG studies in our review examined basic auditory processing and more specialized topics such as music and speech perception. This is partly because passive listening paradigms in auditory research (e.g., basic auditory-evoked response, various oddball paradigms, auditory habituation paradigms) are easier to implement compared with other sensory processing tests. Auditory research is especially suitable for MEG recording, because neural generators inside auditory cortex are tangentially oriented, and the neural currents of this direction are best detected by the MEG sensors [40,94]. Most of the infant MEG studies on basic auditory processing investigated the maturation of auditory-evoked response/field (AER, AEF) to pure or complex tones, mismatch response (MMR) or late discriminative negativity (LDN) to tone-change detection. The experiments were usually carried out in the oddball paradigm [44,45,46,57,58,59,60,61,63], some with block presentations [56,64], and one in auditory habituation paradigm [62]. Only one out of the 12 studies reported source localization results using equivalent current dipoles (ECDs) with spherical head model [57], and another one is examining the orientations of the fitted ECDs [56]. Huotilainen et al. [57] successfully localized ECDs of AER and MMR in most of the newborns’ auditory cortices, verifying the location of the auditory neural generators to repetitive and novel sounds. The majority of the studies focused on the developmental trends of AEFs and recorded invaluable longitudinal data from fetuses to newborns. The basic AEF response rates (exceeded baseline noise) increased as a function of age, and the amplitude and latency of the landmark AEFs (P1m, N2m) usually showed increased and shortened trends, respectively. Even though not all the studies found significant results due to the high variability of the fetal and neonatal magnetic responses (some showed the opposite polarity), the MEG waveform analyses answered the fundamental questions about early auditory processing. These studies also showed that MMR or LDN could be elicited by different non-speech tones in most of the neonates and some of the fetuses, indicating a neural registry of an early form of auditory memory/learning mechanism.

### 2.2. Speech and Music

Infant speech perception experiments have focused on the ability to discriminate more sophisticated sound categories with distinction in temporal structure, phonemic category, or prosody at different ages. Among the ten selected infant speech processing studies (one with music intervention), over half of them incorporated source localization analysis to verify their hypotheses [47,65,67,70,72]. Unlike adult MEG studies in which individual subjects’ MRIs are generally available for source modeling analysis, most of the infant studies used a representative age-appropriate infant head template with spherical head modeling for source estimation. Boundary element method (BEM) is another forward modeling adopted by some of the studies to estimate the signal projections from each cortical source to the MEG sensors other than the spherical head modeling [70,72]. A range of different inverse models were adopted in these studies, including ECD, minimum norm estimate (MNE), standardized low-resolution brain electromagnetic tomography (sLORETA), and dynamic statistical parametric mapping (dSPM), to estimate the locations of the neural generators of the targeted cognitive tasks based on the constraints of the forward models. By incorporating MEG source localization techniques, infant speech studies could elucidate the neuroanatomical underpinnings of cross-language speech categorization [70,72], motor theory in infants [67], and even testing infant’s semantic processing [48] and how music training benefits later speech processing [47] with both temporal and spatial characterizations that other neural imaging tools may not be able to provide. The maturation of MEG measurement, a non-invasive and zero-noise source imaging tool for infants, will add more perspectives to the speech development theories building on infants’ behavioral responses.

### 2.3. Somatosensory Activity

Somatosensory development is also an area that utilized MEG intensively to investigate newborns and older infants’ maturity in sensory processing. The two main types of somatosensory stimuli—tactile and electric stimulations—can be applied while infants are asleep. At the same time, MEG can be applied to record their somatosensory-evoked magnetic fields (SEFs). The setup usually involves a plastic membrane attached to an infant’s index finger, wrist, or back of the palm contralateral to the recorded hemisphere. Recording neurophysiological response during infants’ sleep could minimize the head movement and increase the number of tested trials for more precise source modeling later. Indeed, almost all the infant somatosensory MEG studies incorporated dipole fitting to identify the locations of early and late sensory responses [73,74,75,76,77,78,79]. With abundant infant research on somatosensory-evoked potentials (SEPs) measured by EEG, MEG measurement provides not only the waveform results to corroborate previous electrophysiological findings but also the locations of the neural activities, which were usually not reported in EEG studies. The consistent types of tactile stimulation and generally clear dipole locations make it possible to compare the neural generators of sensory processing across studies. The magnetic signals with a high tolerance of irregular skull structures could avoid confounds due to infants’ physical development. A helpful comparison of dipole locations of the somatosensory responses across ages and studies on common SEF components (e.g., M30, M60, M200, etc.) was provided in the review article by Nevalainen et al. [39]. 

As mentioned earlier, almost all infant somatosensory MEG studies incorporated source localization analysis (except [66]). All of the reports used ECD (single- or multi-dipole) with a spherical head model to estimate the sources of the corresponding brain activity. Meltzoff et al. [79] further applied exact low-resolution brain electromagnetic tomography (eLORETA) to examine the distributed dipole locations of each time point. The MRI structural images were generally not obtained from each infant, but some studies did acquire at least one example of infant MRI and used it to confirm the precision of the dipole fitting [73,77,78]. The noteworthy thing is that the goodness-of-fit of the ECDs was usually above 70% and could reach 97% at peak latency for some individuals in the reported studies (e.g., [75]). Moreover, the ECDs could be successfully modeled in at least half of the infants, sometimes 100% in neonates [77]. Together, the MEG measurements and neural activation sources findings in infant somatosensory processing showed promising results. The development and refinement of this body of research could pose further questions based on the existing knowledge of fundamental sensory processing. For example, Meltzoff et al. [79] explored more sophisticated questions about social cognition in terms of self-other sensory experience, and they found that seven–month-olds’ primary somatosensory cortices were activated when they saw other people’s hands being touched. Another future possibility is to define the typical sensory response in primary and associative somatosensory cortices, and use them as biomarkers to evaluate the neural development of infants at risk. 

### 2.4. Vision

The maturation of visually-evoked responses (VERs) in early development has been tested through habituation paradigm by Matuz et al. [81] and Sheridan et al. [80]. Neither studies applied source localization analysis for the recorded VERs. Since the research focused on characterizing perceptual habituation from fetal to infantile stage, the waveform analysis comparing amplitudes and latencies of the VERs to different stimuli could answer the key research questions adequately. Additionally, the visual habituation studies longitudinally recorded fetuses and newborns, providing an invaluable developmental trajectory of VERs in the context of sensory habituation as an early form of learning.

Future visual studies measuring VERs with MEG can incorporate source analysis to verify the neural generators of fundamental visual processing. The source of VERs elicited by flashes of light should reside approximately in the primary visual cortex in the occipital region. The verification of the location of primary visual processing measured with MEG will provide a baseline for future studies on more sophisticated visual processing (for example, face recognition).

### 2.5. Sleep

Infant brain activation in sleep is probably tied to most of the research themes mentioned above. To reduce influences from movement-related artifacts, many of the infant MEG studies were carried out when the participants were asleep for better data quality. Nonetheless, the numbers of reports examining the relation between sleep stage and neural mechanisms of perception in each sensory domain fell short compared to the reports on each specific perceptual task. The three infant MEG studies on sleep patterns confirmed the waveforms usually seen in EEG recordings in different sleep stages, and the sleep spindles between different states [85,86,87]. The brain activities associated with various sleep stages are characterized by the amplitude and regularity of the continuous waveforms, and also by the rhythm observed from the power spectrum of the brain response. Source analysis has not been reported for infant’s sleep patterns since they are usually non-dipolar except for sleep spindles showing some dipolar distribution around central sulcus [87]. 

The effect of how sleep stage influences evoked response has not been consistently characterized. Some earlier studies did not report different AEFs in newborns’ quiet or active sleep [46,85]. A later study found that late AEFs elicited by short bursts of pure tone (750 ms after the sound onset) were moderately diminished in active sleep in infants younger than four months, but not older infants [86]. The same study applied localization analysis and confirmed the auditory cortical source of AEFs, but the related details were not further reported. Other studies on neonatal AEFs elicited by speech also observed a stronger response in quiet sleep compared to active sleep, but the MMR was similar across the sleep stages [44,66,68]. For SEFs, there were more pieces of evidence of how somatosensory-related components had higher amplitudes in quiet sleep compared to active sleep (e.g., [66,76]). The overall results indicate that sleep stage may impact evoked responses in some degrees, which should be taken into account for future infant MEG experimental designs especially for studies comparing across groups of subjects that are recorded at different alertness levels. Future studies can further investigate whether sleep stage can affect dipole fitting in different perceptual tasks, similar to what Nevalainen et al. [76] and Lauronen et al. [75] presented in their somatosensory studies.

### 2.6. Motor Activity

The same research group, Berchicci et al. [82] and Berchicci et al. [83], conducted two infant motor studies using MEG. The motor research focused on the development of mu rhythm, which presents in the resting stage but is suppressed during the motor movement. Time-frequency analysis was applied to compare the frequency peak of mu rhythm in infancy, childhood, and adulthood. There is an increasing trend of the frequency peak from infancy (around 3 Hz) to adulthood (around 10 Hz), and the increasing rate was the largest in infant’s first year of life (from 3 Hz to 8.25 Hz). Even though no studies included source localization analysis, the consistent dependency of mu rhythm on motor movement suggests a neural generator in sensorimotor areas, which needs to be confirmed in future follow-up studies [82].

### 2.7. Clinical Studies: Epilepsy

Aside from capturing typical cognitive development, MEG has been applied to assist the pre-surgical mapping for the pediatric population with epilepsy (for a review, see Reference [95]). Infants with symptomatic epilepsy experience seizures at a very high frequency, sometimes every few minutes [51]. The determination of the source location of the epileptic activation is of high clinical values [90]. The identification of epileptic foci by MEG recordings is useful for individuals suffering from recurrent seizures whose MRI scans did not reveal lesion parts. The sensitivity of the MEG recordings for mapping out the focus of the epileptic activities adds extra information to the pre-surgical evaluation. The incorporation of MEG with patients’ EEG or MRI records provides more reliable pre-surgical work-up and is associated with better post-surgical outcomes [91]. 

Several studies have successfully implemented MEG source localization in infant epileptic patients using adult MEG helmets [90]. Multi- or single-equivalent current dipoles (ECDs) with individual MRI was the most common way to estimate the focal region of epileptiform activation in each patient. The goodness-of-fit could reach 80% in most cases. Most importantly, a high percentage of epileptic infants went seizure-free after incorporating the epileptic focal identification through MEG recordings in their pre-surgery assessment [91]. The widely used MEG source analysis in pediatric populations, especially in individuals with epilepsy, demonstrated that MEG is a popular non-invasive tool with high temporal and spatial precision when combined with individual MRI scans. On the whole, by only relying on MEG source localization result will not be enough to predict a high successful post-surgical outcome. Integrating multiple imaging tools and obtain concordant locations of epileptic spikes is the most promising way for pre-surgical evaluation and prognosis. 

## 3. Advances and Limitations in Infant MEG Source Localization Analysis

Approximately half of the studies summarized above incorporated source analysis. The successful implementation of infant MEG source localization was reflected in less-constrained dipole models and more advanced head modeling, which overcame the inherent challenges in recording infant neurophysiological activities with adult MEG system. From the signal processing end, the continuous head position measurement and head movement compensation help preserve more trials with deviant head positions, which were usually discarded in the past [50,96,97]. Higher numbers of preserved trials provide a better signal-to-noise ratio (SNR) and lead to more reliable source analysis results.

The precision of the forward modeling increased from using adult head models to estimate the projection of the neural generators, to using infant-size spherical head models. The simplified single-shell head models have been shown to work fine in localizing the neural generators of the fundamental perceptual processing. A more sophisticated way is to take different types of physiological structures into account by using BEM or other realistic head methods for forward modeling [1]. Later studies using templates from a series of age-matched infant MRIs to identify the sources of the cognitive processing have also been shown to be effective when individual infant MRIs were not available [67]. The advancements of the forward modeling methods better configure the parameters for the next source analysis step—the inverse solution. 

The most widely used inverse solution in the infant MEG studies was equivalent current dipole (ECD), with few reports applying sLORETA [70,72] and dSPM [47,48]. Both single- and multiple-dipole fitting have been applied in the current infant MEG literature. Different degrees of constraints on dipole locations and orientations were chosen based on the research questions or the inherent properties of the target neuromagnetic components. The strengths of the dipoles in certain prior-defined regions of interest (ROI) were calculated for a more focused statistical testing [48,70]. In general, the refinement of the inverse solution to locate the neural activities supports a more nuanced way to explain infant cognitive processing and development.

With the advancement of the software development, newer MEG hardware designed for younger populations has emerged to resolve the low SNR issues stemming from the considerable distance between the adult MEG helmet and the infant brain [38]. BabySQUID was first launched with a partial-head coverage, high MEG sensor density, and child-head-size dewar, and it exhibits excellent sensitivity to neonatal MEG signals [98]. The high sensitivity and spatial precision of BabySQUID showed successful SEFs detection from averaging as few as four trials. Another system Artemis 123, part of the BabySQUID family, was also introduced for measuring infant and child’s brains [52]. The later whole-head BabyMEG was even able to detect evoked response in a single-trial via real-time signal processing [52,98,99]. The higher SNR can also help shorten the recording time of the studies that require infant participants to be awake, increasing the success rate of the experiments. Even with its advantage of high spatial precision and SNR, not all the reports using infant-child MEG systems applied source analysis. Future research should leverage the high signal quality of these infant MEG systems for identifying the neural sources of infant cognitive processing.

Even though more verification is still required before applying to infant population, the latest on-scalp MEG showed another possibility to record neuromagnetic signals with relatively less requirement of stillness from the participants [49,100,101,102]. In this case, the distance between the cortex and sensor would be the closest, leading to a high quality of neurophysiological signals. Furthermore, the head movement compensation may no longer be needed. Forward modeling for on-scalp MEG will need more optimization to account for individualized sensor array layout [49].

## 4. Future Directions

The advancement of infant MEG source analysis and its implementation in various cognitive tasks are encouraging, and new research directions for this imaging tool are proposed here. Although recent years have seen the increasing popularity in time-frequency analysis and functional brain connectivity analysis, source estimation of particular neural oscillation has not been examined via MEG yet. For instance, Berchicci et al. [82] and Berchicci et al. [83] studying motor development looked at the maturation of the mu rhythm and found drastic changes in oscillatory peak with age. They speculated that the potential oscillatory source of this motor-related brain rhythm could be pinpointed, which provides a new lens for examining cognitive neural development. The extensive research on primary and secondary somatosensory processing also suggests another possibility to look at the neural connectivity within or perhaps between the sensory processing systems. The establishment of typical patterns of local or long-range functional connectivity will provide invaluable tools for early abnormal brain functioning detection. One concern is that the reliability of functional connectivity should build on multiple recordings from the same individuals [103], which may be more challenging in infant participants. One last call on the technical side is the implementation of real-time analysis, which is supported by BabyMEG system [99], to pediatric epilepsy population. Real-time analysis with stronger signals recorded by child MEG systems can help the online detection of the epileptic activity, and perhaps providing real-time source localization.

Future potential research topics can focus more on multisensory integration across sensory modalities and more naturalistic stimuli (for example, the use of phrases and sentences rather than the use of monosyllabic syllables) for posing more ecologically valid research questions. Meltzoff et al. [79] and Travis et al. [48] have already demonstrated that neural mechanisms underlying cross-sensory integration in infants could be reliably measured and localized with MEG. Research questions in infant cognition can go from bottom-up signal detection or discrimination moving forward to top-down processing by using tasks involved cross-modal matching. Another emerging research theme is how social interaction shapes early development. This topic could be addressed by using hyperscanning, which records both infant and the other person’s brain response when they are engaging in the same task [104]. The social interaction can be further categorized into infant-infant or infant-adult scenarios by using MEG-EEG, MEG-fMRI, or other combinations of different imaging tools. Even though how to integrate multimodal neuroimaging methods needs further verification, the incorporation of multiple advanced imaging tools shows promising trends for future research efforts.

The MEG localization methods for infants and children can be applied to clinical pediatric populations other than epilepsy. For example, individuals with autism spectrum disorder tend to have atypical neural registries of sounds [105]. MEG could record the abnormal neurophysiological components and neural oscillatory responses along with their cortical/subcortical origins, and further propose potential biomarkers for early detection for pre-diagnostic groups (e.g., [106,107]). Preterm infants’ cortical functioning is also of significant concerns. Some somatosensory reports suggested that very preterm infants did not show typical SEFs from primary and secondary somatosensory cortex [39]. Studies focusing on preterm infants’ basic sensory processing will enrich our current knowledge, which is mostly built on typically developing infants. The differences between preterm and full-term infants’ sensory processing will lay the foundation for future studies looking at developmental neural and cognitive functioning in both groups of infants. Other neurological and psychiatric disorders like cerebral palsy, Down syndrome, traumatic brain injuries (TBI), Tourette syndrome, opioid-exposed infants (neonatal abstinence syndrome, NAS), etc., can also leverage the advancement of MEG measurement and source localization [108,109].

Developmental cognitive neuroscience research is on the rise with multiple approaches to gain insights about the developmental trajectories [110]. It is essential to point out that few MEG studies have employed a longitudinal design. Longitudinal studies [58,111] can reveal the developmental trajectory of brain signal variability and complexity from fetus [56,71,112,113,114,115,116] over the entire life span. The importance of longitudinal data cannot be overestimated [117]. Each neuroimaging tool has its advantages and disadvantages. The purpose of this review is not to advocate MEG as the only and best tool to understand early brain development. Instead, we suggest that MEG, with its advanced source estimation, is an informative tool that can be more widely used than the current percentage of reports we have surveyed here. Lastly, the target research questions should drive the selection of the methods. Not all of the unanswered underpinnings of cognitive development require the identification of neural generators. Given the current under-utilized status in infant research, MEG should be considered as one of several imaging techniques that can localize the sources of the brain activations for elucidating the research questions. With the current trends in the neuroscience field moving from genome to connectome, the use of portable devices with more cost-effective and user-friendly measures, and machine learning with the use of big data, further infant MEG research holds the promise to increase our knowledge of the development of normal brain functions and search for biomarkers for the diagnosis and treatment of neurodegenerative and neuropsychiatric disorders.

## Figures and Tables

**Table 1:** A summary of the infant MEG papers reviewed.

Authors (years)	Sample Size	Age(s)	Materials	Paradigms & Components	Recording Parameters	Preprocessing	Head Position Standardization	Source Modeling	No source Analysis, other Analysis
**Auditory**									
Lengle, Chen and Wakai [56]	F: 19; N: 16	F: 20 – 40 weeks; N: 0.5 – 1.5 months	Pure tone	Block; AEF	R side recorded; 96 trials in each run, 4 – 8 runs	Spatial & matched filter; Manual artifact rejection; Average; Bandpass filter (2–10 Hz)	Not reported	Not reported	Waveform analysis
Huotilainen, Kujala, Hotakainen, Shestakova, Kushnerenko, Parkkonen, Fellman and Näätänen [57]	12	2–12 days	Tones with 2 upper harmonics	Oddball; MMR	Either side recorded; Accepted at least 350 standard trials & 95 deviant trials	Epoch (−150–700 ms); Artifact rejection (> 1500 fT/cm); Average; Bandpass filter (1–20 Hz); Baseline correction; SSP	Verify at the beginning of each recording	ECD with spherical head model	
Cheour, Imada, Taulu, Ahonen, Salonen and Kuhl [46]	4/8	1–6 days	Tones with 3 upper harmonics	Oddball; MMR	L side recorded;	Epoch (−100–700 ms); Head movement rejection; SSS; Lowpass filter (20 Hz); Baseline correction;	Translated to a reference head location of the device coordinate system	Not reported	Waveform analysis
Holst, Eswaran, Lowery, Murphy, Norton and Preissl [58]	F: 16/18; N: 14/18	F: above 27 weeks; N: 6 days–6 weeks	Pure tones	Oddball; AEF	Both sides recorded;	SSP; Epoch (−200–800 ms); Artifact rejection (> 2 pT); Average; Bandpass filter (0.5–10 Hz)	Not reported	Not reported	Waveform analysis
Draganova, Eswaran, Murphy, Huotilainen, Lowery and Preissl [59]	F: 12; N: 5	F: 33–36 weeks; N: < 0.5 months	Tones with 2 upper harmonics	Oddball; MMR & LDN	Supine position; Accepted at least 300 standard trials & 44 deviant trials	SSP; Epoch (−100–600 ms); Artifact rejection (> 2 pT); Average; Bandpass filter (0.5–10 Hz)	Not reported	Not reported	Waveform analysis
Sambeth, Huotilainen, Kushnerenko, Fellman and Pihko [45]	12/13	1–8 days	Tones with 2 upper harmonics	Double oddball; MMR & LDN	R side recorded; Accepted at least 100 trials	Movement artifact rejection; Average; SSS; Vector sums; Lowpass filter (40 Hz)	Recorded but not standardized	Attempted but not reported	Waveform analysis
Draganova, Eswaran, Murphy, Lowery and Preissl [60]	F: 18; N: 9	F: 28–29 weeks, follow-up every 2 weeks	Tones with 2 upper harmonics	Oddball; MMR & AEF	Supine position; Accepted at least 600 standard trials & 70 deviant trials	SSP; Artifact rejection (> 2 pT); Average; Bandpass filter (0.5–10 Hz)	Not reported	Not reported	Waveform analysis
Sambeth, Pakarinen, Ruohio, Fellman, van Zuijen and Huotilainen [44]	12/13	1–8 days	Tones with 2 upper harmonics	Multifeature oddball; MMR, LDN, & AEF	R side recorded; Accepted at least 140 trials	Epoch; Artifact rejection; SSS (for 2 infants); Vector sums; Lowpass filter (40 Hz)	Recorded but not standardized	Not reported	Waveform analysis
Sheridan, Draganova, Ware, Murphy, Govindan, Siegel, Eswaran and Preissl [61]	F: 20/22; N: 15	F: 29–38 weeks; N: 2–38 days	Pure tones	Oddball; AEF	Supine position	SSP; Epoch (−200–1000 ms); Or epoch (−100–800 ms); Artifact rejection (> 2 pT); Average	Not reported	Not reported	Waveform analysis
Muenssinger, Matuz, Schleger, Kiefer-Schmidt, Goelz, Wacker-Gussmann, Birbaumer and Preissl [62]	F: 36/41; N: 15/22	F: 30–39 weeks; N: 6–89 days	Pure tones	Auditory habituation; AEF & MMR	R side recorded	SSP; Highpass filter (1 Hz); Lowpass filter (N: 15 Hz); Epoch (−90–330 ms); Artifact rejection (> 2 pT)	Not reported	Not reported	Waveform analysis
Schleger, Landerl, Muenssinger, Draganova, Reinl, Kiefer-Schmidt, Weiss, Wacker-Gußmann, Huotilainen and Preissl [63]	F: 23/30; N: 16/30	F: 30–39 weeks; N: 14–89 days	Pure tones	Oddball; MMR	R side recorded	SSP; Bandpass filter (N: 1–15 Hz); Epoch (−200–750 ms); Artifact rejection (> 2 pT)	Not reported	Not reported	Waveform analysis
Edgar, Murray, Kuschner, Pratt, Paulson, Dell, Golembski, Lam, Bloy and Gaetz [64]	29/36	6–59 months	Pure tones	Block; AEF (P2m, N2m)	Accepted trials ranged from 30 to 206	Downsampled (300 Hz); Epoch (−200–400 ms); Artifact rejection; Average; Bandpass filter (2–55 Hz)	Recorded but not standardized	Not reported	Waveform analysis
**Music**									
Zhao and Kuhl [47]	71/94	9 months	Piano and woodblock sounds; Synthesized speech /bi/	Oddball; MMR	Presented 1250 trials (200 deviant trials)	tSSS; Head movement compensation; SSP; Bandpass filter (1–40 Hz); Remove bad channels; Epoch (−50–900 ms); Artifact rejection (> 2 pT/cm, or peak-to-peak > 1.5 pT); Average; Baseline correction	Aligned to individual mean head position; Source space and the BEM surface aligned and scaled to fit individual head shape	BEM isolated-skull approach with inner skull surface from an MRI template; dSPM without dipole orientation constraints	
**Speech**									
Kujala, Huotilainen, Hotakainen, Lennes, Parkkonen, Fellman and Näätänen [65]	10	1–25 days	Vowels	Oddball; MMR	Either or both sides recorded	Epoch(−150–700 ms); Artifact rejection (> 1500 fT/cm); Average; SSP; Bandpass filter (1–20 Hz); Baseline correction	Recorded but not standardized	ECDs with spherical head model with origin (0, 0, 25) mm	
Pihko, Lauronen, Wikström, Taulu, Nurminen, Kivitie-Kallio and Okada [66]	10/18	1–4 days	Single syllables	Oddball; MMR & AEF (P1m, P2m)	R side recorded	Average; Vector sums; Lowpass filter (40 Hz)	Not reported	Not reported	Waveform analysis
Imada, Zhang, Cheour, Taulu, Ahonen and Kuhl [67]	N: 18; 6-month: 17; 12-month: 8	5 days; 6 months; 12 months	Pure tones; Harmonics; Single syllables	Oddball; MMR	L side recorded	Epoch (−100–800/1200 ms); Head movement rejection; SSS; Average; Head standardization; Lowpass filter (20 Hz); Baseline correction	L auditory regions aligned to have the same position and orientation	MNE L1 based on ROIs with spherical head models (1 for each age)	
Sambeth, Ruohio, Alku, Fellman and Huotilainen [68]	11	1–5 days	Singing; Speech	Alternating blocks; AEF (P1m)	R side recorded; Accepted at least 125 trials	Epoch (−100–800 ms); Head movement rejection; Average; SSS; Vector sums; Lowpass filter (40 Hz)	Not reported	Not reported	Waveform analysis
Bosseler et al. [69]	6-month: 7; 12-month: 11; Exclude 17 infants; Adult: 9	6 months; 12 months; Adult	Single syllables	Oddball; Theta oscillation	Whole-head measurement	Epoch (−100–1200 ms); Average; tSSS; Head movement compensation	Converted to a standardized position within the MEG sensor array	Not reported	Time-frequency analysis
Kuhl, Ramírez, Bosseler, Lin and Imada [70] Exp. 1	7-month: 7/25; 11-month: 10/24; Adult: 10/14	7 months; 11 months; Adult	Single syllables	Double oddball; MMR	Whole-head measurement; Accepted at least 40 trials	SSS; tSSS; Head movement compensation; Artifact rejection (peak-to-peak > 8 pT/cm); Average; Lowpass filter (20 Hz); Baseline correction	Recorded but not reported	MNE with spherical head model, using 6-mo MRI template	
Kuhl, Ramírez, Bosseler, Lin and Imada [70] Exp. 2	7-month: 8; 11-month: 8; Excluded: 16	7 months; 12 months	Single syllables	Same as Exp. 1	Whole-head measurement; Accepted at least 30 trials	SSS; Bandpass filter (1–20 Hz); SSP; tSSS; Head movement compensation; Artifact rejection (peak-to-peak > 1.5 pT/cm); Average; Baseline correction	Recorded but not reported	BEM isolated-skull approach with inner skull surface from 12-mo MRI template, and sLORETA without dipole constraints	
Hartkopf et al. [71]	F: 30; N: 28	F: 28–39 weeks; N: 0–3 months	Single syllables	Auditory habituation; AEF	R side recorded; 140 sequences each with 8 trials	SSP; Lowpass filter (F: 10 Hz, N: 15 Hz); Highpass filter (1 Hz); Epoch (−100–500 ms); Artifact rejection (> 2 pT)	Not reported	Not reported	Waveform analysis
Ferjan Ramírez, Ramírez, Clarke, Taulu and Kuhl [72]	16/33	11 months	Single syllables	Double oddball; MMR	Whole-head measurement; Accepted at least 75 trials	Downsampled (500 Hz); tSSS; Head movement compensation; SSP; Bandpass filter (1–40 Hz); Epoch (−100–700 ms); Artifact rejection (> 3 pT/cm or 4 pT); Average; Baseline correction	Transformed to the mean head position; Source space and the BEM surface aligned and scaled to fit individual head shape	BEM with 14-mo MRI template, and sLORETA without dipole constraints	
**Somatosensory**									
Gondo, Tobimatsu, Kira, Tokunaga, Yamamoto and Hara [73]	12	12–18 months	Air pressure pulses	Block; SEF	R side recorded; Accepted 128 or 256 trials	Epoch (−50–250 ms)	Not reported	Single ECD with spherical head model	
Pihko, Lauronen, Wikström, Taulu, Nurminen, Kivitie-Kallio and Okada [66]	6/14	1–3 days	Air pressure pulses	Block; SEF (P1m, P2m)	R side recorded	Average; Movement rejection; SSS	Not reported	Not reported	Waveform analysis
Pihko, Lauronen, Wikström, Parkkonen and Okada [74]	16	1–5 days	Electrical stimulation; Air pressure pulses	Block; SEF (M30, M70, M250)	R side recorded	Average; Movement rejection; SSS; Bandpass filter	Not reported	Single ECD	
Lauronen, Nevalainen, Wikström, Parkkonen, Okada and Pihko [75]	N: 26; 6-mo: 5; Adult: 10	N: CA 38–42 weeks; 6-months: 6.5 months; Adult	Electrical stimulation; Air pressure pulses	Block; SEF (N1, N20m)	R side recorded; Accepted trials ranged from 92 to 267	Epoch (start from −100 ms); Average; Movement rejection; SSS; Bandpass filter (1–90 Hz)	Recorded but not reported	ECDs with spherical head model	
Nevalainen, Lauronen, Sambeth, Wikström, Okada and Pihko [76]	20/21	1–6 days	Air pressure pulses	Block; SEF (M60, M200)	R side recorded; Accepted 250 trials	Epoch (start from −100 ms); Average; SSS or SSP; Baseline correction; Lowpass filter (90 Hz)	Recorded but not reported	ECDs with spherical head model with origin (0, 0, 30) mm	
Pihko, Nevalainen, Stephen, Okada and Lauronen [77]	51 (20, 9, 8, 8, 12)	1 d–57 years (Newborn, 6-months, 12–18 months, 1.6–6 years, Adult)	Air pressure pulses	Block; SEF (M30, M60)	R side recorded; Accepted trials ranged from 106 to 686	Epoch (start from −100 ms); Average; SSS; tSSS; Bandpass filter (1–90 Hz)	Recorded but not reported	ECDs with spherical head model with origin from individual’s preauricular and nasion crossing	
Nevalainen, Pihko, Metsäranta, Sambeth, Wikström, Okada, Autti and Lauronen [78]	44/46	1–23 days	Air pressure pulses	Block; SEF	R side recorded, some with both sides recorded; Accepted 265 trials on average	tSSS; Movement artifact rejection; Epoch (start from −100 ms); Average; Lowpass filter (90 Hz)	Recorded but not reported	ECDs with spherical head model	
Meltzoff, Ramírez, Saby, Larson, Taulu and Marshall [79]	Exp 1: 21/30; Exp 2: 22/41	7 months	Air pressure pulses; Videos of hands being touched	Block; SEF	Whole-head measurement; 400 or 480 air pulses trials, and 44 or 50 for video trials	tSSS; Head movement compensation; SSP; Bandpass filter (1–40 Hz); Head position standardization; Epoch (−250–750/1750 ms); Artifact rejection (> 3 pT/cm or 4 pT)	Transformed to individual’s mean head position; Later transformed to the mean head position of all infants	ECD, eLORETA with 3 dipoles at each time point	
**Vision**									
Sheridan, Preissl, Siegel, Murphy, Ware, Lowery and Eswaran [80]	25 (follow up this group)	F: 29–37 weeks; N: 6–22 days	Light flashes	Short-term habituation; VER	Occipital region recorded; 60 or 90 sequences each with 4 flashes	SSP; Epoch (−1000–1000 ms); Artifact rejection (> 2 pT); Average	Recorded but not reported	Not reported	Waveform analysis
Matuz, Govindan, Preissl, Siegel, Muenssinger, Murphy, Ware, Lowery and Eswaran [81]	F: 37/40; (follow up some of them) N: 23/26	F: 30–38 weeks; N: 6–22 days	Light flashes; Pure tone	Short-term habituation; VER	Occipital region recorded; 90 sequences each with 4 flashes followed by a tone	SSP; Epoch (−1000–1000 ms); Artifact rejection (> 2 pT); Average	Recorded but not reported	Not reported	Waveform analysis
**Motor**									
Berchicci, Zhang, Romero, Peters, Annett, Teuscher, Bertollo, Okada, Stephen and Comani [82]	I: 14/25; C: 12/18; A: 6	I: 11–47 weeks; C: 24–60 months; A: 20–39 years	Grasp or squeeze a pipette	Block; Mu rhythm	L side recorded; Accepted 20 trials	Artifact and 60 Hz line noise removal; Artifact rejection (manual); Functional topography approach; Bandpass filter (0–10 Hz for infants)	Recorded but not standardized	Not reported	Time-frequency analysis
Berchicci, Tamburro and Comani [83]	I: 14/25 C: 12/18 A: 6	I: 11–47 weeks C: 24–60 months A: 20–39 years	Grasp or squeeze a pipette	Block; Mu rhythm	L side recorded	Bandpass filter (0.5–40 Hz); PCA; ICA reject artifact	Not reported	Not reported	Time-frequency analysis
**Multimodal**									
Travis, Leonard, Brown, Hagler Jr, Curran, Dale, Elman and Halgren [48]	16/24	12–18 months	Spoken words; Signal corrected noise; Pictures	Block; N400m	Whole-head measurement; 30 trials of each condition	Lowpass filter (50 Hz); Bad channel removal; Artifact removal (> 3000 fT/cm); ICA artifact removal; Epoch (−200–1200/1500 ms)	Recorded but not reported	BEM and dSPM with cortex reconstructed from individual MRI	
Pihko et al. [84]	22	1–18 days	Air pressure pulses; Vowels	Alternating stimuli; AEF, SEF	L side recorded; Accepted trials ranged from 75 to 596	Epoch (start from −100 ms); Average; Movement artifact rejection; tSSS; Lowpass filter (90 Hz); Baseline correction	Recorded but not reported	ECDs with spherical head model	
**Sleep**									
Lutter, Wakai, Maier and Baryshnikov [85]	7	1.5–8.5 weeks	Pure tone; Sleep	Block; AEF, Sleep patterns	Not reported	Not reported	Not reported	Not reported	Waveform analysis
Lutter, Maier and Wakai [86]	10/18	CA 39–66 weeks	Pure tone; Sleep	Block; AEF, Sleep patterns	Accepted at least 60 trials	Bandpass filter (0.5–20 Hz)	Recorded 3 participants but not reported	ECD fitted but not reported	Waveform analysis
Wakai and Lutter [87]	7	CA 46–63 weeks	Sleep	Sleep patterns, Sleep spindles	R side recorded	Lowpass filter (30 Hz)	Not reported	No reported	Time-frequency analysis
**Spontaneous**									
Haddad et al. [88]	19/21	CA 38–45 weeks	Awake or sleep	Spontaneous pattern	Both sides and back position recorded	Cardiac artifact rejection (manual); Highpass filter (0.5 Hz); Lowpass filter (70 Hz);	Not reported	Not reported	Continuous waveform analysis;Time-frequency analysis
**Epilepsy (examples)**									
Hanaya et al. [89]	19	0.5–14 years	Epileptic spikes; Total intravenous anesthesia	Resting	Whole-head measurement in supine position; Recording time ranged from 10 to 38 minutes	Bandpass filter (10–70 Hz); Notch filter (60 Hz)	Not reported	Single moving dipole with single-shell spherical model	
Shibata, Mosher, Kotagal, Gupta, Alexopoulos and Burgess [90]	9	< 2 years	Epileptic spikes	Resting	Whole-head measurement in supine position; Averaged recording time 62 minutes	tSSS; Head movement compensation (to initial head position)	Shifted to default position	Single ECD with spherical head model	
Shukla, Kazutaka, Gupta, Mosher, Jones, Alexopoulos and Burgess [51]	9	10 months–15 years	Epileptic spikes	Resting	Whole-head measurement in supine position; Averaged recording time 58 minutes; Averaged 38 spikes	tSSS	Recorded	Single ECD coregistering to individual MRI	
Garcia-Tarodo, Funke, Caballero, Zhu, Shah and Von Allmen [91]	31	3–23 months	Epileptic spikes	Resting	Recording time ranged from 60–75 minutes;	Not reported	Recorded but not reported	Multiple ECD	
*Acronyms	Number of “included/ recruited” samples; F = fetuses; N = neonates; I = infants; C = children; A = adults; Exp = experiment	CA = conceptional age		AEF = auditory-evoked field; MMR = mismatch response; LDN = late discriminative negativity; SEF = somatosensory-evoked response; VER = visually-evoked response;	R = right; L = left	t/SSS = temporal signal-spaceseparation; SSP = signal-space projection; PCA = principal component analysis; ICA = independent component analysis		ECD = equivalent current dipole; BEM = boundary element methods; dSPM = dynamic statistical parametric mapping; e/sLORETA = exact/standardized low-resolution electromagnetic tomography; MNE = minimum norm estimation; ROI = regions of interest; MRI = magnetic resonance imaging	

## Abbreviations

AER/F	Auditory-Evoked Response/Field
BEM	Boundary Element Method
dSPM	dynamic Statistical Parametric Mapping
ECD	Equivalent Current Dipole
EEG	Electroencephalography
eLORETA	exact Low-Resolution Brain Electromagnetic Tomography
EOG	Electro-oculogram
FEM	Finite Element Method
fMRI	functional Magnetic Resonance Imaging
fNIRS	functional Near-Infrared Spectroscopy
HPI	Head Position Indicator
ICA	Independent Component Analysis
LDN	Late Discriminative Negativity
MEG	Magnetoencephalography
MMR	Mismatch Response
MNE	Minimum Norm Estimation
MRI	Magnetic Resonance Imaging
MSP	Magnetically Shielded Room
MSI	Magnetic Source Imaging
NAS	Neonatal Abstinence Syndrome
NIRS	Near-Infrared Spectroscopy
PET	Positron Emission Tomography
PCA	Principal Component Analysis
ROI	Region of interest
SEF	Somatosensory-Evoked Field
SEP	Somatosensory-Evoked Potential
sLORETA	standardized Low-Resolution Brain Electromagnetic Tomography
SNR	Signal-to-Noise Ratio
SPECT	Single-Photon Emission Computed Tomography
SQUID	Superconductive Quantum Interference Devices
SSP	Signal-Space Projection
SSS	Signal-Space Separation
TBI	Traumatic Brain Injuries
tSSS	temporal Signal-Space Separation
VER	Visually-Evoked Response
